# Reconstruction of a 2D layer of KBr on Ir(111) and electromechanical alteration by graphene

**DOI:** 10.3762/bjnano.12.35

**Published:** 2021-05-11

**Authors:** Zhao Liu, Antoine Hinaut, Stefan Peeters, Sebastian Scherb, Ernst Meyer, Maria Clelia Righi, Thilo Glatzel

**Affiliations:** 1 Department of Physics, University of Basel, 4056 Basel, Switzerland; 2 Department of Physics and Astronomy, University of Bologna, 40127 Bologna, Italy

**Keywords:** DFT, graphene, Ir(111), KBr, KPFM, nc-AFM, surface reconstruction

## Abstract

A novel reconstruction of a two-dimensional layer of KBr on an Ir(111) surface is observed by high-resolution noncontact atomic force microscopy and verified by density functional theory (DFT). The observed KBr structure is oriented along the main directions of the Ir(111) surface, but forms a characteristic double-line pattern. Comprehensive calculations by DFT, taking into account the observed periodicities, resulted in a new low-energy reconstruction. However, it is fully relaxed into a common cubic structure when a monolayer of graphene is located between substrate and KBr. By using Kelvin probe force microscopy, the work functions of the reconstructed and the cubic configuration of KBr were measured and indicate, in accordance with the DFT calculations, a difference of nearly 900 meV. The difference is due to the strong interaction and local charge displacement of the K^+^/Br^−^ ions and the Ir(111) surface, which are reduced by the decoupling effect of graphene, thus yielding different electrical and mechanical properties of the top KBr layer.

## Introduction

Many two-dimensional (2D) materials have excellent optical, mechanical, electromagnetic, and other attractive physical properties [1–2]. Their structures can be usually tailored or modified at the nanoscale [3–4]. One type of modification is the fabrication of 2D layered heterostructures, which have been the subject of intensive scientific research in recent years [5–7]. The topological insulator Bi_2_Se_3_ can be used, for example, for the fabrication of 2D layered heterostructures with graphene. Thus, systems can be created to study the unusual interaction of Dirac fermions, opening up new possibilities for novel electronic and spintronic devices [5]. Another famous 2D insulator is hexagonal boron nitride (h-BN), which cannot only be used as a functional insulating monolayer [8–10], but can also be stacked with graphene layers [11–12] and has, among other interesting properties, a very stable structural superlubricity [7]. Besides the stacking of different monolayers, the intercalation of a third material between a substrate and a 2D layer can be applied to tune the physical properties [13–17]. Schulzendorf et al. reported the electrical decoupling of graphene islands by the intercalation of single layers of KBr between the Cu(111) metal substrate and graphene, resulting in quasi free-standing graphene layers [18].

Alkali halide layers are frequently used as decoupling layers in surface science [19–22]. They are reported to form single- or double-layer islands with a typical cubic structure on single-crystalline transition metal surfaces [23–24]. Compared to the adsorption energy, the ionic interaction within the layer is typically stronger such that structural deformations are weak [25]. However, it was demonstrated that a NaCl adlayer on Ag(100), besides strongly reducing the overall work function, can create clearly modulated dipole moment densities corresponding to the registry of the NaCl layer in respect to the substrate [26–27]. But also the formation of a weak structural deformation observed as superstructure has been reported for (100) oriented surfaces, for example, for KCl and NaCl on Ag(100) [28–29] or KBr on Cu(100) [30]. In contrast, on hexagonal (111) oriented metal surfaces, alkali halides were reported to arrange in cubic islands [31–33]. Sometimes, they exhibit a moiré pattern, for example, as a result of the incommensurate growth in the system of NaCl/Cu(111) [34]. Furthermore, considering the interface of alkali halide heterostructures, the top KBr film on NaCl(100) exhibits a superstructure with square islands, while the stretched NaCl film on KBr(100) grows flat with rectangular islands, clearly indicating a stronger interaction between the adlayer and the substrate in these examples [35]. In contrast to these studies, a completely different possibility of a reconstruction for NaCl was recently predicted theoretically, suggesting that increasing the interaction between substrate and film by selecting a particular substrate may lead to the formation of NaCl films with unusual structures [36]. Such 2D NaCl layers with unconventional stoichiometries have been observed indirectly on graphene surfaces [37].

Here, we report on the formation of irregularly shaped KBr islands with corrugated stripe structures, observed on the (111) surface of Ir and analyzed by non-contact atomic force microscopy (nc-AFM) at room temperature and density functional theory (DFT) calculations. The results suggest that this particular reconstruction of KBr occurs on Ir(111), due to a specific correlation of the lattice parameter. When deposited on a single layer of graphene on the same substrate, the topography of the KBr islands returns to the expected cubic configuration of bulk KBr and the work function of the system is strongly altered.

## Results and Discussion

**Figure 1:**
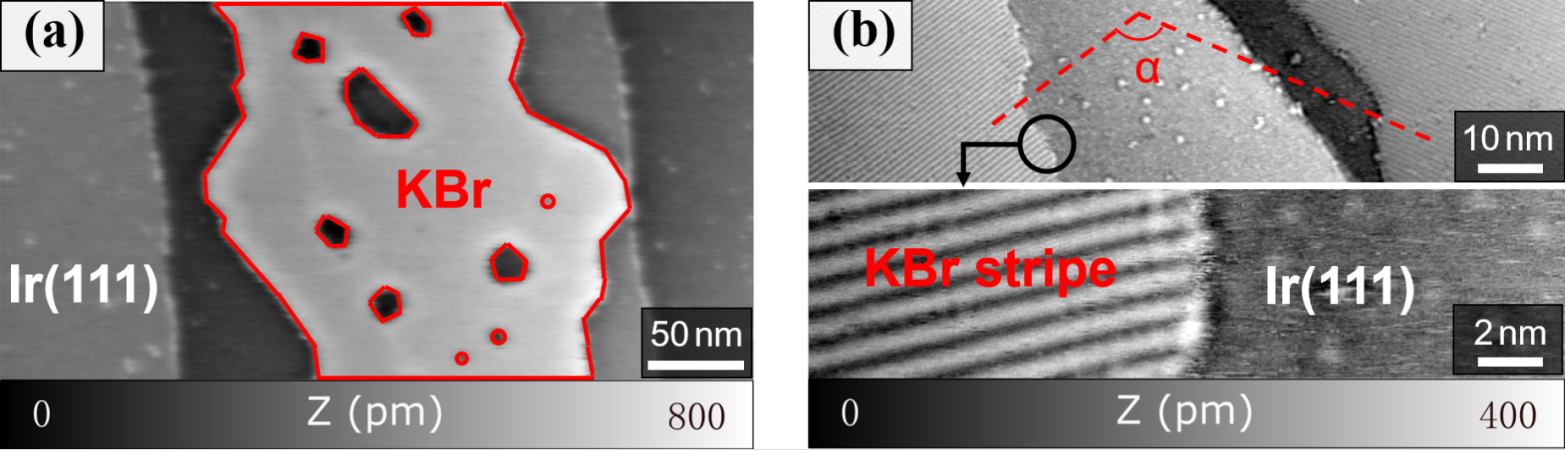
Reconstructed KBr on the Ir(111) surface. (a) Large-scale KBr island with irregular shape (*A*_1_ = 5 nm, Δ*f*_1_ = −4 Hz, γ = −13.7 pN·nm^1/2^). (b) Two different KBr domains with clear line structure and a zoom-in at one of the KBr edge regions (*A*_1_ = 2 nm, Δ*f*_1_ = −25 Hz, γ = −21.6 pN·nm^1/2^).

The thermal deposition of less than a monolayer of KBr on an atomically clean Ir(111) surface under ultrahigh vacuum (UHV) conditions results in the formation of islands on the iridium terraces. Figure 1a shows a large-scale topography image of a KBr island on Ir(111) measured by nc-AFM at room temperature. The monoatomic steps between the Ir terraces have a height of 235 pm as expected for Ir(111). The KBr islands are monolayers with an average height of 340 pm under the compensated bias voltage of −430 mV, presenting several holes and fuzzy edges. Remarkably, the growth of the islands mainly occurs at the middle of the Ir terraces, rather than at step edges as observed, for example, for NaCl on Ag(111) [33] or graphene (GR) on Ir(111) [38–39]. Also, the irregular and round shape is far from the expected cubic island structure. More details of the internal structure of the KBr islands are visible in the magnified measurements presented in Figure 1b. The structure of the islands is dominated by stripes running through the whole island with a periodicity of about 1.1 nm. The analysis of multiple islands reveals that three different orientations of the line structures are equally observed on the surface with specific orientations of 120° as shown in Figure 1b and Supporting Information File 1, Figure S1a.

**Figure 2:**
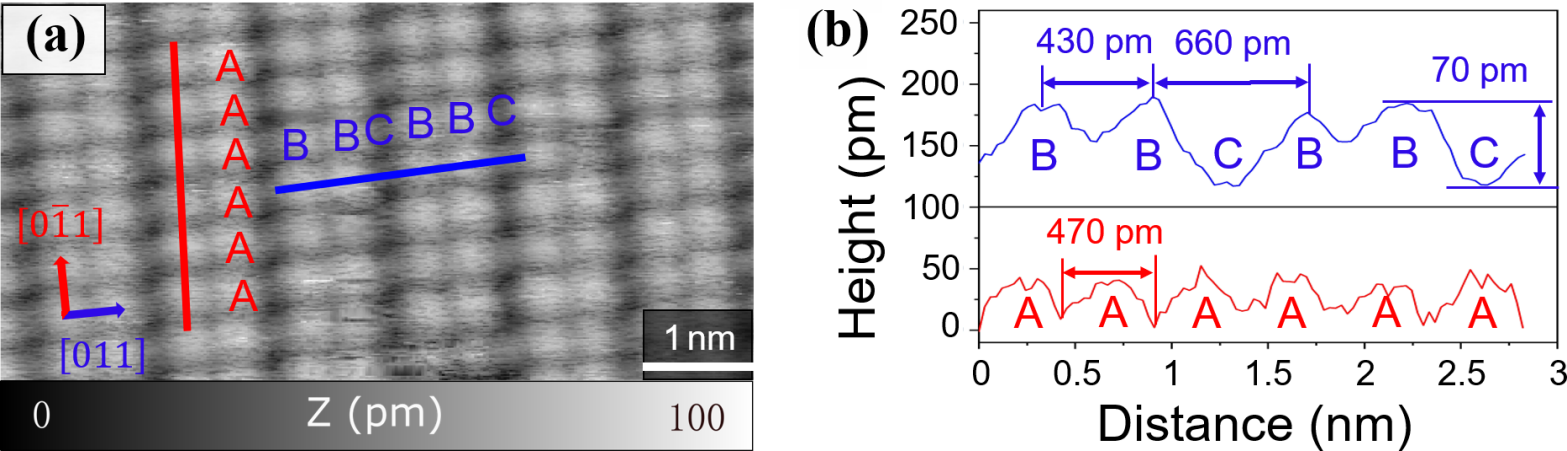
(a) Atomic resolution of a corrugated KBr line structure (*A*_2_ = 300 pm, Δ*f*_2_ = −125 Hz, γ = −1.02 pN·nm^1/2^). (b) Atomic line profiles of two orthogonal lattice directions in red (with a single-atom duplication along “AAA”) and in blue (with a triple-atom duplication along “BBC”) indicated in (a), respectively.

A closer look at the atomic scale of these structures is presented in the high-resolution image in Figure 2a. The line structures consist of two atomic-scale protrusions separated by a valley. Two types of orthogonal lattice arrangements are observed, that is, one with a regular single-atom repetition along the stripes (red line) and another with a triple-atom repetition orthogonal to the lines (blue mark). The atoms along the stripe lines with the “AAA” pattern are separated by 470 pm on average, which is in the range of both the nearest-neighbor distance between two equal ions of bulk KBr in the 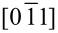 direction (
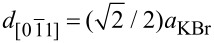
 = 466 pm) [40] and the distance of the long diagonal of the iridium unit cell (
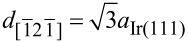
 = 470 pm) [41]. This observation already corresponds well with the three directions observed for the line structures and the main crystallographic directions of the Ir(111) surface. However, in the orthogonal direction (i.e., [011] orientation), the lattice arrangement of KBr is rather unexpected with a repetition of “BBC”, where “B” stands for the top atoms and “C” for the dark valley between the double lines. The distances were determined to be, on average, 430 pm and 660 pm for “BB” and “BCB”, respectively. Obviously, the combination of KBr and Ir(111) favors a new surface structure, different from the one on other metal substrates such as Cu(111) [42]. Now, the question arises if this structure is a moiré lattice (lateral relaxation) induced by an alternating strength of interaction forces or a new reconstruction of the KBr layer.

To search for this answer, density functional theory (DFT) calculations have been performed to conclude on the observed structure. As a fundamental consideration, the lattice match for the 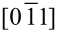 orientation of KBr to fit the 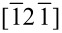 direction of Ir(111) due to 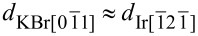 was used and several possible periodicities have been considered as presented in Supporting Information File 1, Figure S2. Initially, a system with a periodicity of Ir(111) along the 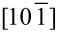 direction of 21 × *a*_Ir_ and along 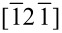 of 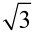 × *a*_Ir_ (21 × 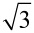) was considered, resulting in a moiré pattern similar to the experimental observations. The structural optimization in a variable cell of this system and of slightly modified ones (21 × 2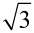, 12 × 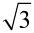, and 12 × 2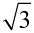) gave the best results in terms of system stability only for modified structural arrangements of the KBr layer. Clusters of three KBr units are periodically formed to minimize the overall adsorption energy. The calculated adsorption energy for the 12 × 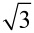 and for the larger 12 × 2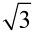 configuration is in both cases −5.31 eV/nm^2^ (−0.82 eV/unit, the adsorption energy in a unit cell) and thus stronger than on Cu(111), where it was −2.43 eV/nm^2^ [18]. Calculation of the specific adsorption energies of K and Br and their distance from the Ir surface at the equilibrium geometry revealed that fcc or hcp threefold hollow sites are more favorable for the adsorption. While the K^+^ ions are almost at the equilibrium adsorption distance, the Br^−^ ions are strongly corrugated.

**Figure 3:**
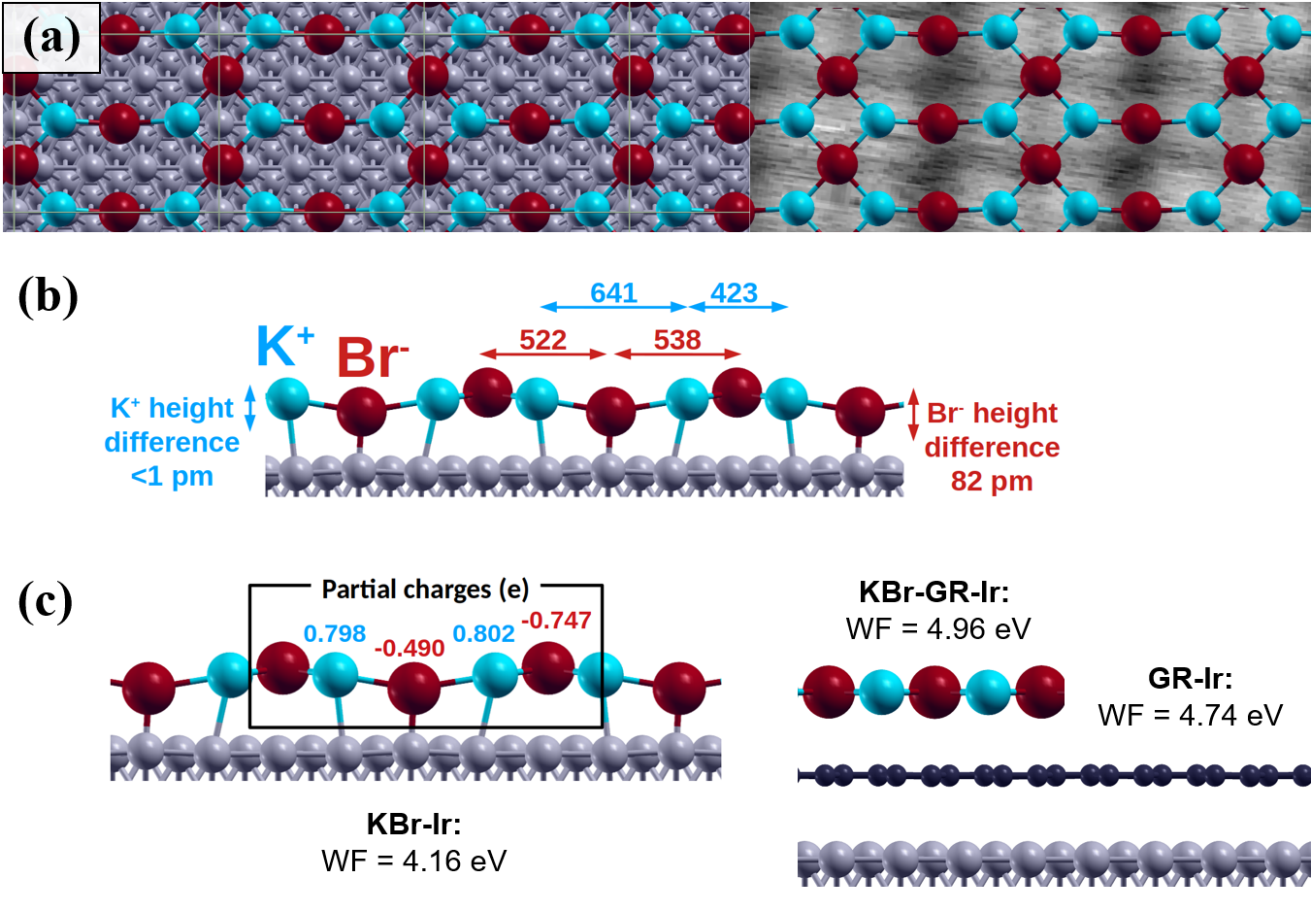
DFT simulation of a monolayer KBr on Ir(111). (a) Optimization of a reconstructed KBr layer on Ir(111) with a cell size of 4 × 2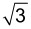 superimposed on the Ir(111) model and experimental data (Br^−^ in red and K^+^ in blue, the size of the atoms reflects their ionic radii). The interaction with the substrate induces a strong corrugation and distortion of the KBr layer, as shown in the side view in (b). (c) Partial charges and resulting work function (WF) of KBr on Ir(111), of KBr on GR/Ir(111), and of only graphene on Ir(111). For the corrugated KBr layer a significant decrease of work function is observed, while the work function increases when a graphene layer is present between KBr and Ir(111).

Although the earlier proposed models already give a first impression of the special interaction and reconstruction of this system, the experimental results are not reproduced in all details. Therefore, a periodic 4 × 2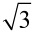 cell was utilized including such a cluster to relax and optimize the structure presented in Figure 3. The structure is built up by repeating KBr clusters forming the double-row structure connected by Br^−^ ions, which are more strongly bound to the Ir substrate. Figure 3a shows a top view of the optimized structure superimposed to a part of the experimental image. The side view in Figure 3b details the atomic distances along the structure of 641 pm between the rows and 423 pm in the rows measured for the K^+^ ions along the [011] direction and of 461 pm in the 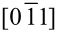 direction. The height variation of the K^+^ ions is only about 1 pm and that of the Br^−^ ions is 82 pm, which agrees well with the experimental observations. The adsorption energy is calculated to be −4.63 eV/nm^2^, which is per KBr unit (−0.91 eV/unit) lower than the one for the models discussed above (see Supporting Information File 1, Figure S2). With this knowledge, the observed structure can be assigned to a novel reconstructed KBr configuration, and the bright protrusions in the AFM measurements can be assigned to be a signature of the potassium ions. Also, to support the stability, it is found more favorable for Br^−^ ions to shift their positions to reach a lower-energy adsorption state. Additionally, the three observed KBr domains (Figure 1 and Figure S1, Supporting Information File 1) are a result of the overlap with the axis of Ir(111) every 120°.

Furthermore, a Bader charge analysis [43–46] was performed to evaluate the partial charges of the reconstruction as shown in Figure 3c. The positive charge of K^+^ stays almost constant while the negative charge of the Br^−^ ions is modulated by the distance to the Ir(111) substrate, inducing an overall reduction of the work function to Φ_DFT(KBr/Ir(111))_ = 4.16 eV. Taking bare Ir(111) as a reference with a calculated work function of Φ_DFT(Ir(111))_ = 5.76 eV, experimentally a work function of Φ_EXP(KBr/Ir(111))_ = 4.06 eV was measured by Kelvin probe force microscopy (KPFM), as can be seen in Supporting Information File 1, Figure S3.

**Figure 4:**
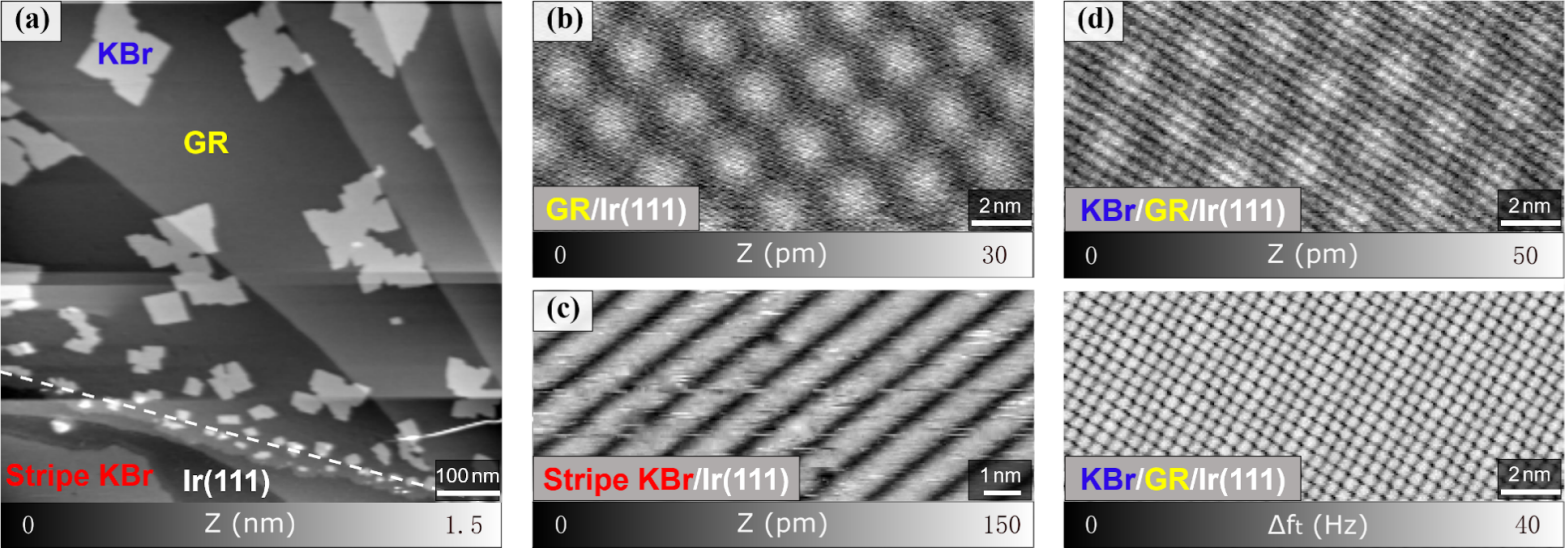
Cubic KBr on the GR/Ir(111) surface. (a) Two different KBr structures have been discovered on GR/Ir(111) and bare Ir(111). (b) Single moiré pattern of GR/Ir(111) (*A*_1_ = 2 nm, Δ*f*_1_ = −85 Hz, γ = −70 pN·nm^1/2^). (c) Corrugated KBr on bare Ir(111) with the stripe structure (*A*_1_ = 2 nm, Δ*f*_1_ = −50 Hz, γ = −41 pN·nm^1/2^). (d) Multiple moiré pattern of GR/Ir(111) (*A*_1_ = 2 nm, Δ*f*_1_ = −300 Hz, γ = −247 pN·nm^1/2^), with the torsional frequency shift Δ*f_t_* image below (*A_t_* = 30 pm).

To be able to tune this corrugated structure, a monolayer of graphene was prepared on Ir(111) before KBr deposition. Figure 4a shows a large-area topography of the Ir(111) surface, half of which is covered by graphene and additionally less than one monolayer of KBr. For Cu(111), KBr intercalation, which induces a decoupling of the graphene layer from the metal substrate, was observed with a similar preparation [18]. Here, different types of KBr patterns are found in separated regions with and without graphene. In the regions of bare Ir(111), the islands described above can still be observed with the stripe structure as seen in the lower left corner of Figure 4a and the magnified view in Figure 4c. However, square islands are formed on the Ir(111) surface covered with graphene. Additionally, there are some small KBr clusters at the edge of the graphene sheets as indicated by the white dashed line in Figure 4a. A clear moiré pattern is observed on the bare graphene surfaces on Ir(111) in high-resolution measurements as presented in Figure 4b and as expected for GR/Ir(111) [47–49]. However, different from the reconstructed stripe structures on Ir(111), the square-shaped KBr islands on the single layer of graphene recover a simple cubic structure as shown in Figure 4d. The upper figure shows the topography of such an island while the lower figure presents the simultaneously measured torsional frequency shift, which is related to short-range interaction forces and highlights, therefore, the atomic periodicity [50]. The topography shows not only the cubic KBr lattice but also the hexagonal graphene moiré, which shines through the KBr layer and has dimensions (2.42 nm) similar to those of the pure GR/Ir(111) system. The lattice constant of the KBr layer is determined to be 651 pm, in accordance to the expected 660 pm for bulk KBr. In comparison to the KBr growth mode on bare Ir(111), as presented in Figure 1a, the KBr layer on graphene is observed to even extend across step edges. Hence, the reconstructed KBr islands with stripe lines seem to be a unique phenomenon on the Ir(111) surface. A single layer of graphene below KBr interrupts this special reconstruction and alters it back to the cubic arrangement as also shown by our DFT calculations (Figure 3c). These calculations furthermore show that the KBr layer is almost flat when graphene is intercalated and that the K^+^ and Br^−^ ions are equally polarized, that is, their partial charges tend to compensate. The measured work function of this structure (Supporting Information File 1, Figure S3) is increased compared to bare KBr (Φ_Exp(KBr/Ir(111))_ = 4.06 eV) and to the single layer of graphene (Φ_Exp(GR/Ir(111))_ = 4.56 eV) on Ir(111). Experimentally, a value of Φ_Exp(KBr/GR/Ir(111))_ = 4.71 eV is determined, which is in reasonably good agreement with the DFT value of Φ_DFT(KBr/GR/Ir(111))_ = 4.96 eV (Figure 4c), taking into account the effects of tip contamination and averaging effects in KPFM [51].

## Conclusion

In conclusion, a novel corrugated reconstruction of KBr was observed on Ir(111) by nc-AFM and confirmed by DFT simulations. It is attributed to the lattice symmetry of KBr with respect to the iridium substrate in one direction and a self-adjustment in the orthogonal direction forming characteristic stripe patterns. The DFT simulations verify these alternating structures and predict repeating KBr clusters that form a double-row structure connected by Br^−^ ions, which are more strongly bound to the Ir substrate, while the K^+^ ions keep stable. The work function of this layer is reduced by −1.7 eV compared to Ir(111) as shown by KPFM and in accordance with charge density calculations. A single layer of graphene between KBr and Ir(111) weakens the strong KBr/Ir interaction and the KBr islands recover to the expected cubic lattice. The work function difference compared to the bare Ir(111) surface is reduced to −1.05 eV by the graphene interlayer. Our studies show that already tiny geometric changes, such as, for example, for the formation of KBr adlayers on Cu(111) or Ir(111), a lattice constant difference of only 50 pm leads to a completely different surface structure (e.g., cubic or stripe-like) and, with this, also to a strong change in physical surface properties, such as the work function. The in-depth understanding of such processes might allow us to tune and build up more complex 2D layered systems and adapt them to a specific application.

## Methods

### Sample preparation

The Ir(111) single crystal (MaTeck GmbH, Germany) was cleaned by alternating cycles of Ar^+^ sputtering and annealing at 1400 K under ultrahigh vacuum (UHV) conditions with a base pressure of less than 1 × 10^−10^ mbar. Graphene was prepared by dosing ethylene with a chamber pressure of 2 × 10^−9^ mbar onto the clean Ir(111) substrate at 1300 K for 30 s via a nozzle directly placed above the sample. A KBr bulk crystal with a purity of 99.9% was ground to powder and then thermally evaporated at 700 K with a rate of 0.1 Å/min onto the Ir(111) surface kept at room temperature (300 K).

### Atomic force microscopy

Experiments were performed by using a custom-built UHV AFM microscope operating at room temperature and a base pressure of 5 × 10^−11^ mbar. All images were scanned with silicon cantilevers equipped with sharp tips (PPP-NCL, Nanosensors) running in noncontact AFM mode with a constant amplitude and controlled by the frequency shift. Bimodal AFM was used to combine the first flexural resonance (frequency of *f*_1_ ≈ 165 kHz, amplitude of *A*_1_ = 2–8 nm and a typical quality factor of *Q*_1_ = 30,000) or the second flexural resonance (frequency of *f*_2_ ≈ 1 MHz, amplitude of *A*_2_ = 200–800 pm and a typical quality factor of *Q*_2_ = 10,000) with the torsional resonance detection (frequency of *f*_t_ ≈ 1.5 MHz, amplitude of *A*_t_ = 20–80 pm and a typical quality factor of *Q*_t_ = 100,000) [50,52]. Kelvin probe force microscopy (KPFM) was performed in FM-KPFM mode by applying a DC compensation and AC excitation bias to the sample [51]. The frequency of the excitation was set to *f*_AC_ = 210 Hz and the amplitude to *U*_AC_ = 700 mV, while the oscillation amplitude of the frequency shift Δ*f*_1_(*f*_AC_) was compensated by controlling the applied DC voltage.

### Computational methods

DFT calculations were performed within the local density approximation, using the Perdew–Zunger (PZ) [53] approximation to describe the exchange–correlation functional. The calculations were performed using periodic supercells and the pseudopotential/plane-waves computational approach implemented in the Quantum ESPRESSO computational suite [54–55]. The plane-wave expansion of the electronic wave function (charge density) was truncated using a 30 Ry (240 Ry) cutoff for the kinetic energy, as the pseudopotentials employed in this work were ultrasoft. We added a Gaussian smearing of 0.002 Rydberg to better describe the occupation of the electronic states of the metal around the Fermi energy. A 12 × 12 × 12 K-point grid was used to sample the Brillouin zone of the iridium bulk and the K-point grid was proportionally resized for all the following calculations. We chose not to add any dispersion correction, consistently with our previous investigations [56–57], because these corrections often overestimate the adsorption energies on metallic substrates [58–59]. The process of geometry optimization was stopped when the total energy and the forces converged under thresholds of 1 × 10^−4^ Ry and 1 × 10^−3^ Ry/bohr, respectively.

The adsorption energies *E*_ads_ of the KBr layers on iridium were calculated as follows:

[1] $$E_{\text{ads}}=E_{\text{tot}}-E_{\text{KBr}}-E_{\text{Ir}},$$

where *E*_tot_, *E*_KBr_, and *E*_Ir_ are the energies of the whole system composed by the KBr layer adsorbed on the iridium slab, of the KBr layer alone, and of the iridium slab alone, respectively. To evaluate the adsorption energies of the K^+^ (Br^−^) ions on Ir, an energy difference equivalent to the one shown in Equation 1 was performed, where the total energy of the isolated K (Br) atoms was considered instead of *E*_KBr_.

To calculate the partial charges on the atoms in the simulation cells, the Bader method was chosen [43–46]. To evaluate these charges, Bader identifies the atomic volume as the spatial region delimited zero flux surfaces, that is, the two-dimensional surfaces where the charge density is a minimum perpendicular to the surface. The charge enclosed in the Bader volume can be considered the total electronic charge of the corresponding atom. Therefore, to perform the Bader charge analysis, one needs the whole charge-density grid based on the optimized electronic structure.

To calculate the work functions of the KBr layer adsorbed on Ir, we created a symmetric system, in which both the top and the bottom surfaces of Ir were covered by KBr, and relaxed its geometry. In this way, the planar average of the electrostatic potential energy *V*(*z*) in the vacuum region is completely flat. By taking the difference between the values of *V*(*z*) in the vacuum region and inside the material, the work function is obtained.

The XCrySDen software was used to represent the computational systems [60].

## Supporting Information

File 1 Additional experimental data.
