# Supplementary material for: Reconstruction of a 2D layer of KBr on Ir(111) and electromechanical alteration by graphene
Source: Beilstein J Nanotechnol. 2021 May 11;12:432–9. doi: 10.3762/bjnano.12.35 (PMC8144921; doi:10.3762/bjnano.12.35)
Supplement: File 1 — Additional experimental data. [file Beilstein_J_Nanotechnol-12-432-s001.pdf]

BEILSTEIN JOURNAL OF NANOTECHNOLOGY

Supporting Information

for

Reconstruction of a 2D layer of KBr on Ir(111) and electromechanical alteration by graphene

Zhao Liu, Antoine Hinaut, Stefan Peeters, Sebastian Scherb, Ernst Meyer, Maria Clelia Righi and Thilo Glatzel

Beilstein J. Nanotechnol. **2021,** *12,* 432–439. doi:10.3762/bjnano.12.35

Additional experimental data

License and Terms: This is a supporting information file under the terms of the Creative Commons Attribution License (https://creativecommons.org/licenses/by/4.0). Please note that the reuse, redistribution and reproduction in particular requires that the author(s) and source are credited and that individual graphics may be subject to special legal provisions.
The license is subject to the *Beilstein Journal of Nanotechnology* terms and conditions: (https://www.beilstein-journals.org/bjnano/terms)

After additional calculations, the cell that remains most representative contains 12 Ir and 7 KBr units along the x direction (horizontal direction in the slides). Indeed the mismatch between the theoretical lattice parameters of KBr and Ir within this cell (−1.98%) is very similar to the experimental one of −1.86% in the cell containing 21 Ir units. Moreover, the energy of the relaxed structure in this cell is the lowest among all considered. So we may refer to this structure for a possible model of the "local" system geometry and partial charges.

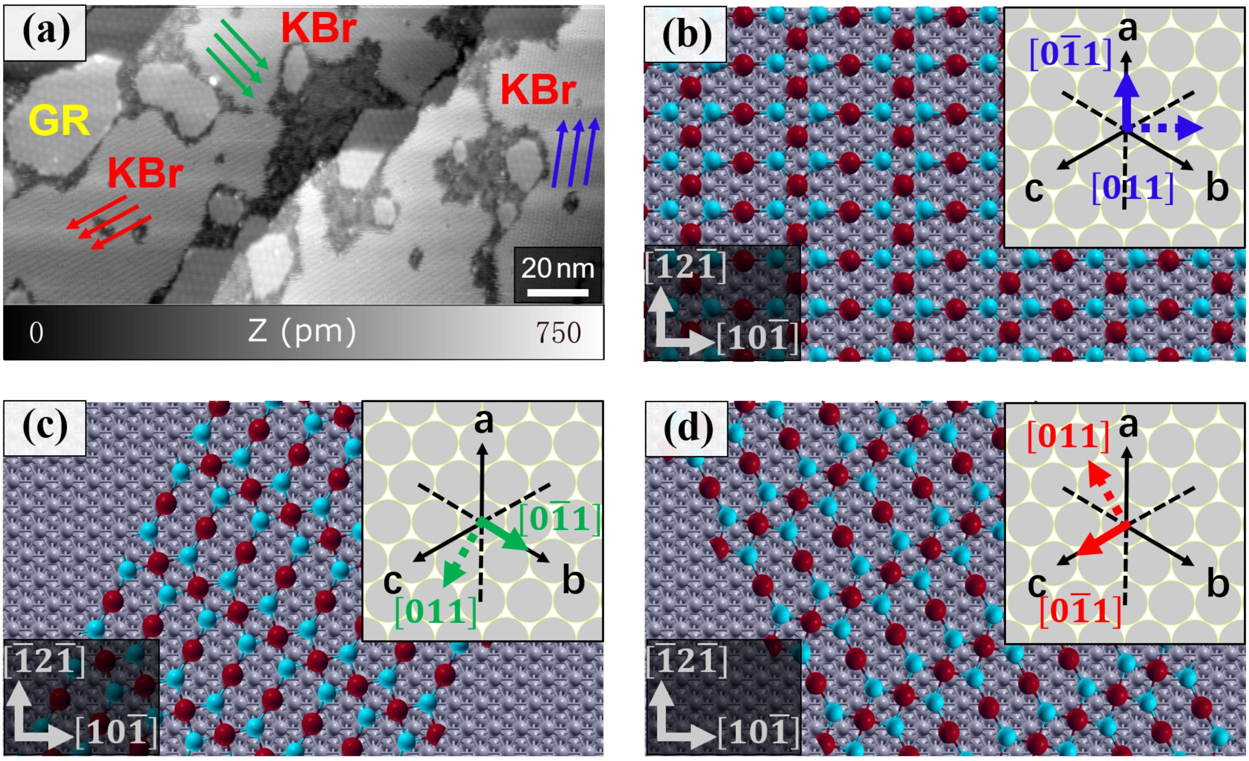

Figure S1: Three rotated domains of the reconstructed KBr(100) lattice with the related models (inset orthogonal arrows with the solid line for [0$\bar{1}$1] (orientation without corrugation) and dotted line for [011] (orientation with corrugation) on hexagonal Ir(111) surface (red for Br atoms, blue for K atoms and grey for iridium atoms)). (a) Topography image of three KBr orientations on Ir(111) in total via nc-AFM ($A_1 = 2\,\text{nm}$, $\Delta f_1 = -30\,\text{Hz}$, $\gamma = -25.9\,\text{pN}\,\text{nm}^{1/2}$). (b) Model in 0° rotation. (c) Model in 120° rotation. (d) Model in −120° rotation.

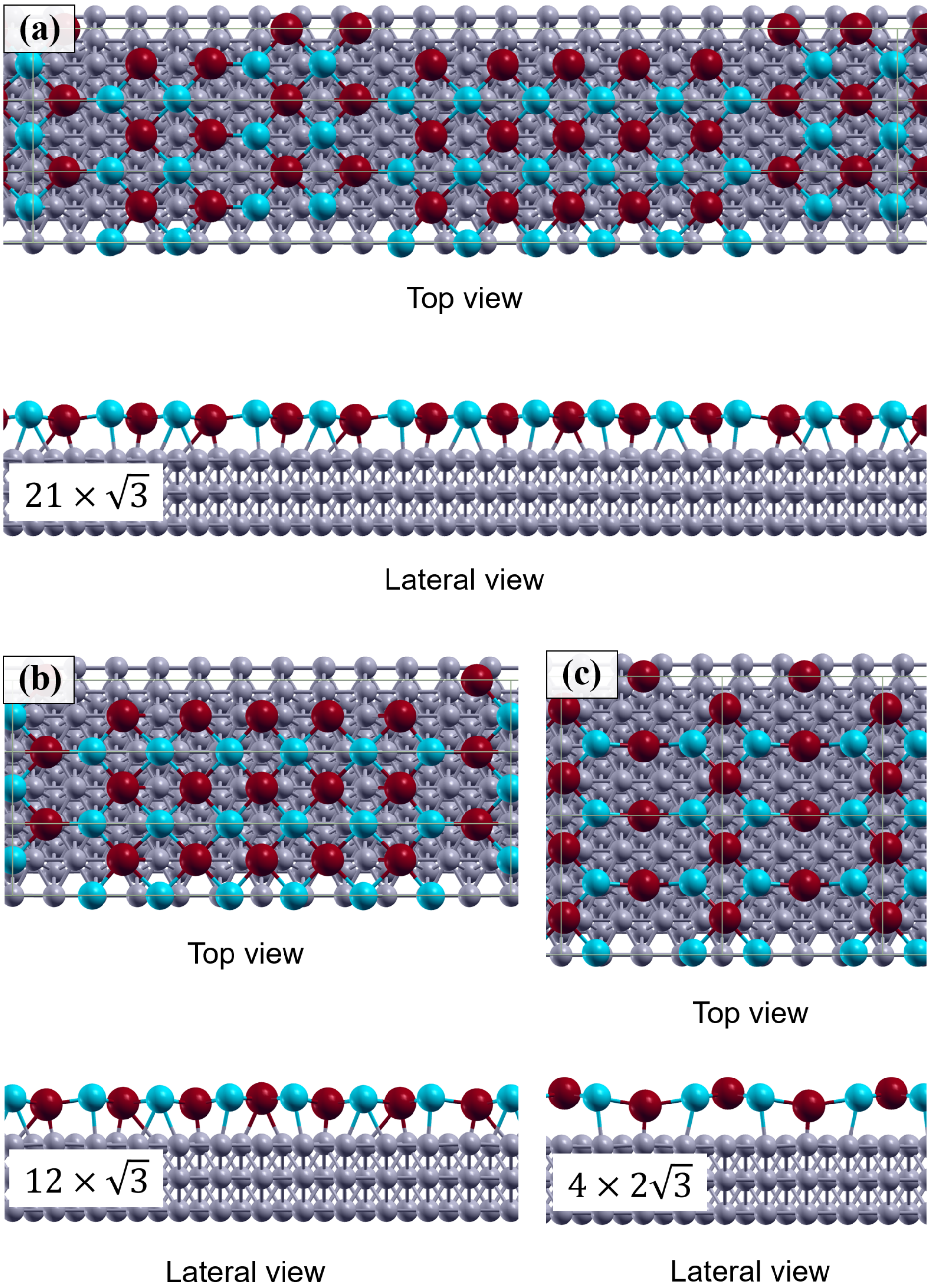

Figure S2: Three calculated KBr reconstructed models on Ir(111) by DFT method from long to short periodicities (red for Br atoms, blue for K atoms and grey for iridium atoms). (a) $21 \times\sqrt{3}$, repeated three times along the $[0\bar{1}1]$ direction, with the adsorption energy of $-0.46\,\mathrm{eV/unit}$. (b) $12 \times\sqrt{3}$, repeated three times along the $[0\bar{1}1]$ direction, with the adsorption energy of $-0.82\,\mathrm{eV/unit}$. (c) $4 \times 2\sqrt{3}$, repeated two times along the $[0\bar{1}1]$ direction, with the adsorption energy of $-0.91\,\mathrm{eV/unit}$.

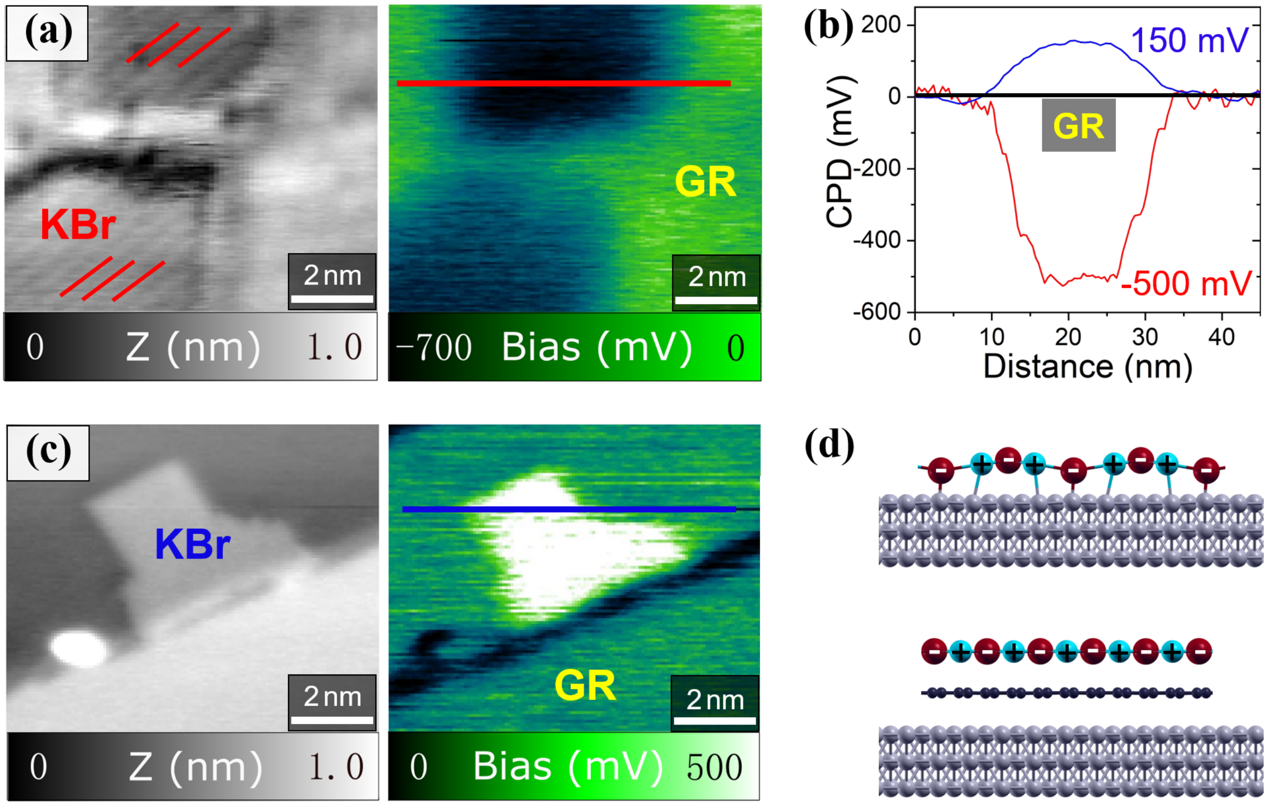

Figure S3: KPFM images of KBr islands accompanied with topography in nc-AFM. (a) KBr island next to the graphene flake (A_1 = 2 nm, Δf_1 = −15 Hz, γ = −12.9 pN nm$^{1/2}$). (b) CPD profiles of two KBr in (a) and (c) compared with graphene. (c) KBr island on the graphene sheet (A_1 = 5 nm, Δf_1 = −5 Hz, γ = −17.1 pN nm$^{1/2}$). (d) The separated charge layers in the reconstructed KBr and the electrically neutral state in the cubic KBr (red for Br atom, blue for K atom and grey for iridium atom).
